# RANKL/RANK/MMP-1 Molecular Triad Contributes to the Metastatic Phenotype of Breast and Prostate Cancer Cells *In Vitro*

**DOI:** 10.1371/journal.pone.0063153

**Published:** 2013-05-16

**Authors:** Sandra Casimiro, Khalid S. Mohammad, Ricardo Pires, Joana Tato-Costa, Irina Alho, Rui Teixeira, António Carvalho, Sofia Ribeiro, Allan Lipton, Theresa A. Guise, Luis Costa

**Affiliations:** 1 Clinical and Translational Oncology Research Unit, Instituto de Medicina Molecular, Faculdade de Medicina de Lisboa, Lisbon, Portugal; 2 Histology Unit, Instituto de Medicina Molecular, Faculdade de Medicina de Lisboa, Lisbon, Portugal; 3 Department of Medicine, Division of Endocrinology, Indiana University, Bloomington, Indiana, United States of America; 4 Pennsylvania State Hershey Medical Center, Hershey, Pennsylvania, United States of America; 5 Oncology Department, Hospital de Santa Maria, Centro Hospitalar Lisboa Norte, Lisbon, Portugal; China Medical University, Taiwan

## Abstract

The osteolytic nature of bone metastasis results from a tumor-driven increased bone resorption. Bone remodeling is orchestrated by the molecular triad RANK-RANKL-OPG. This process is dysregulated in bone metastases, mostly via induction of RANKL by tumor-derived factors. These factors increase expression of RANKL, which induce osteoclast formation, function, and survival, thereby increasing bone resorption. RANK is unexpectedly expressed by cancer cells, and the activation of RANKL-RANK pathway correlates with an increased invasive phenotype. To investigate the interaction between RANK expression in human breast and prostate cancer cells and their pro-metastatic phenotype we analyzed the activation of RANKL-RANK pathway and its effects on cell migration, invasion, gene expression *in vitro*, and osteolysis-inducing ability *in vivo*. RANKL activates kinase signaling pathways, stimulates cell migration, increases cell invasion, and up-regulates MMP-1 expression. *In vivo*, MMP-1 knockdown resulted in smaller x-ray osteolytic lesions and osteoclastogenesis, and decreased tumor burden. Therefore, RANKL inhibition in bone metastatic disease may decrease the levels of the osteoclastogenesis inducer MMP-1, contributing to a better clinical outcome.

## Introduction

Two of the most prevalent cancers, breast and prostate, show high rates of relapse in bone. Between 65% and 75% of patients with breast or prostate cancer will suffer from bone metastases and skeletal-related events (SREs), decreasing the five-year survival by almost 75% and severely increasing morbidity [1], [2]. The osteolytic or osteoblastic phenotypes of bone metastases are caused by an unbalanced bone remodeling, where the tumor cells stimulate both osteoclast (OC) and osteoblast (OB) activity by enriching the tumor microenvironment with tumor-produced factors. In either cases as a consequence of bone remodeling, growth factors are released from bone matrix to further promote tumor growth, in a “vicious cycle” [3], [4].

Bone remodeling is mainly orchestrated by the molecular triad RANK-RANKL-OPG. Receptor activator of NFκB (RANK) is a transmembrane protein that belongs to the tumor necrosis factor receptor (TNFR) superfamily. It is expressed primarily on the cells of the monocytes/macrophage lineage and present on the surface of OCs [5], [6]. RANK ligand (RANKL) is a member of the TNF superfamily [7], expressed by cells of the osteoblastic lineage and lymphoid tissue. Upon RANKL binding to RANK it activates a cascade of intracellular signaling events leading to OC activation, via a TNF receptor associated factor 6 (TRAF6)-dependent signal transduction pathway [8], [9]. Osteoprotegerin (OPG) is another member of the TNFR superfamily that lacks transmembrane and cytoplasmatic domains and is released in soluble form by stromal cells and OBs. Although expressed in several tissues, OPG has a known biological role only in bone, where it inhibits the differentiation and activity of OCs by acting as a decoy receptor for RANKL [10]. RANKL and OPG are dysregulated in a wide range of tumors and bone metastases, mostly via induction of RANKL by tumor-derived factors such as parathyroid hormone-related protein (PTHrP) and tumor-associated suppression of OPG production or activity [11], [12].

RANK is expressed *in vitro* by several human prostate and breast cancer cell lines, including LNCaP, PC-3, Du145, MDA-MB-231, Hs578T and ZR75-1 [13], [14], [15], [16]. RANKL induces the activation of MAPK pathways including ERK1/2 and JNK, and migration and invasion of malignant epithelial cells expressing RANK [13], [14], [17], [18]. It was also demonstrated that RANK pathway is involved in the development of mammary stem cells and breast cancer, promoting tumor initiation, progression and metastasis in human mammary epithelial cells by inducing stemness and epithelial mesenchymal transition [19], [20], [21]. *In vivo*, the expression of RANK by tumor cells has not been widely assessed and results present some discrepancies, either between the percentages of positive samples reported in each study, or according to the prognostic value of RANK expression [13], [22], [23], [24], [25], [26], [27].

Although osteolysis of mineralized collagenous bone matrix by cancer cells involves an increased osteoclastogenesis, it is not yet established whether cancer cells can directly degrade bone. During tumor metastasis, matrix metalloproteinases (MMPs) contribute to angiogenesis, invasion, migration, and final colonization of the metastatic site [28], [29]. MMPs are mainly produced by stromal cells in the surrounding area of tumor cells but many studies demonstrate that MMPs may be up-regulated in tumor cells. Collectively, MMPs are responsible for the bio-available forms of several factors essential in bone metabolism and tumor development and involved in the “vicious cycle” such as TGF-β and RANKL among others. Among MMPs, MMP-1 was identified as one of a small set of causal genes overexpressed in highly bone-metastatic clones of the breast cancer cell line MDA-MB-231 [30], that has a functional role in induction of osteoclastogenesis [31]. MMP-1 is also an important prognostic marker, and we previously reported that serum MMP-1 levels are significantly associated with overall survival of patients with bone metastases [32].

In this work we investigated the interaction between RANK expression in human breast and prostate cancer cells and cell migration and invasion. We found that RANKL stimulates cell migration and invasion through a type I collagen matrix. Upon RANKL stimuli the levels of MMP-1 are increased, while RANK knockdown prevented the MMP-1 up-regulation. RANKL up-regulates *MMP-1* by activating ERK/cFos and JNK/cJun pathways and the MMP-1 promoter. *In vivo*, MMP-1 knockdown significantly decreased osteoclast recruitment to tumor bone interface, leading to a decrease in bone resorption and consequent decrease in bone osteolysis, smaller x-ray osteolytic lesions, and a decrease in tumor burden. These results suggest that if tumor-secreted MMP-1 contributes to the metastatic phenotype of breast and prostate cancer cells, and is regulated by RANKL-RANK pathway, there may be a novel opportunity for the indirect targeting of MMP-1 in bone metastases by targeting the bone microenvironment, namely RANKL.

## Results

### RANKL-RANK pathway mediates migration and invasion of breast and prostate cancer cells

**Figure 1:**
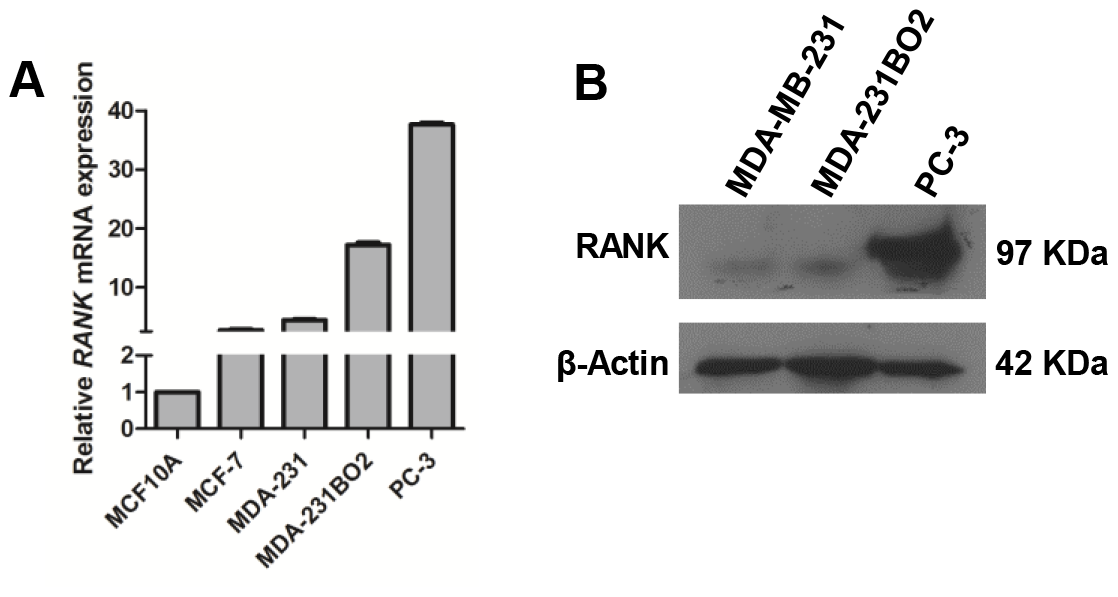
Breast and prostate cancer cell lines express RANK (Receptor Activator of NFκB). *RANK* expression was analyzed by RT-qPCR (a). Protein lysates from MDA-MB-231, MDA-231BO2 and PC-3 cells were analyzed for RANK protein expression by Western blot. β-Actin was used as loading control (b). All experiments were run in triplicate. Error bars represent variation between technical replicates (n = 3).

To assess the putative stimulatory effect of RANKL in RANK-expressing (RANK^+^) breast and prostate cancer cells, three breast cancer cell lines with different metastatic abilities, MCF-7, MDA-MB-231, and MDA-231BO2, and the osteolytic prostate cancer cell line PC-3 were analyzed for RANK expression by RT-qPCR (Fig. 1a). All the cell lines expressed *RANK* mRNA. Results were confirmed at the protein level by Western blot (Fig. 1b). The prostate cancer cells PC-3 had the highest expression of RANK, both at mRNA and protein level, followed by the bone-seeking cell line MDA-231BO2, a clone isolated from MDA-231 cells with increased avidity for bone *in vivo* [33]. Therefore, PC-3 and MDA-231BO2 cell lines were used to investigate the effect of RANK-RANKL pathway in their metastatic phenotype.

**Figure 2:**
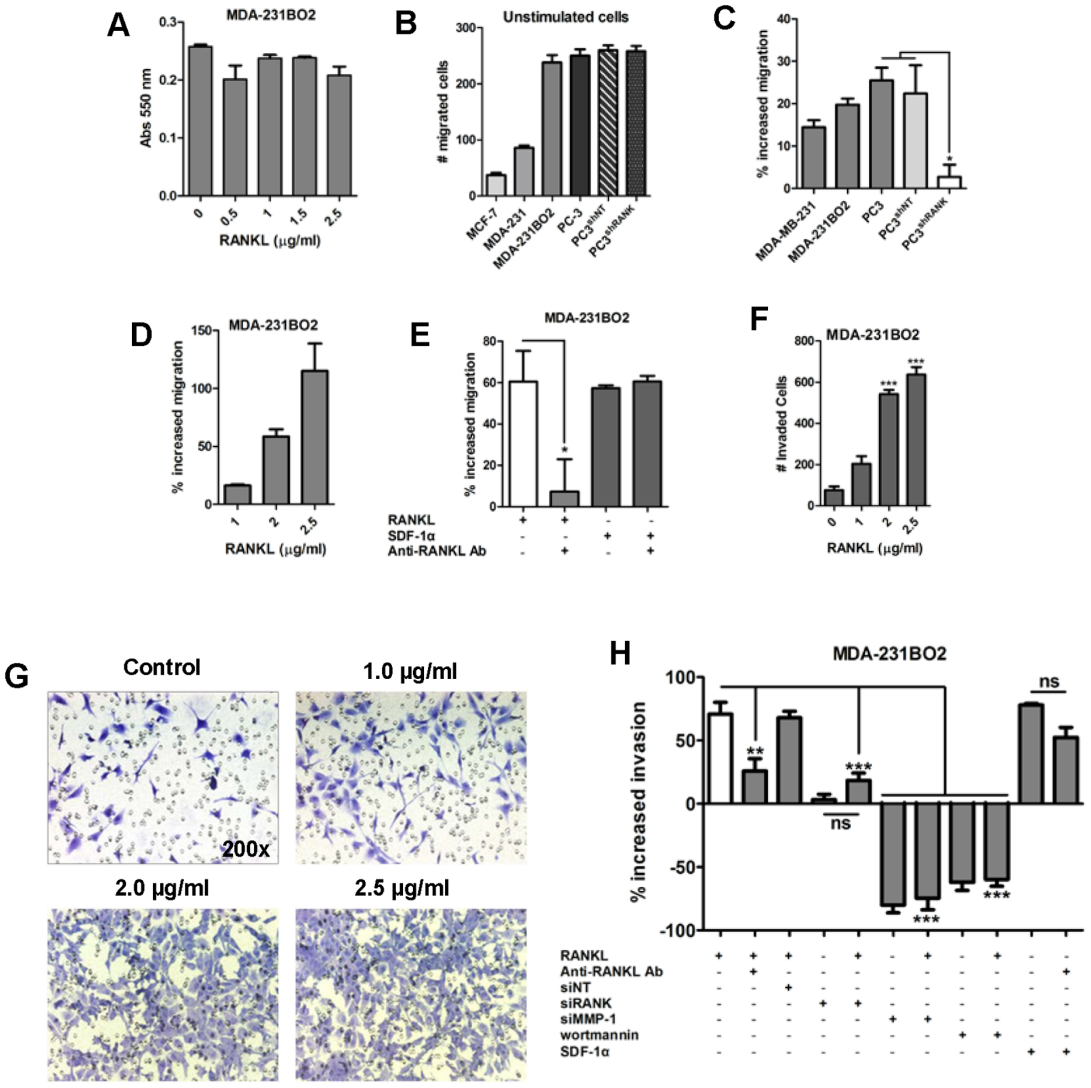
RANKL-RANK pathway mediates migration and invasion of breast and prostate cancer cells. RANKL stimulus does not affect the proliferation of MDA-231BO2 breast cancer cells (a). Migration (b–e) and invasion (f–h) assays were performed. Migration assays were performed with Oris Cell Migration Assay (b–d) or using 96-well chemotaxis chambers with polycarbonate filters (8 µm pore size) (e). Breast and prostate cancer cell lines have different basal migration levels (b). RANKL (1 µg/ml) increases migration of MDA-MB-231, MDA-231BO2 human breast cancer cells and PC-3 human prostate cancer cells, while migration of PC-3^KDRANK^ cells in response to RANKL is significantly decreased. PC-3^shNT^ prostate cancer cells were used as control (c). RANKL increases migration of MDA-231BO2 cells in a dose-dependent manner (d). Increased migration of MDA-231BO2 cells in response to RANKL (2 µg/ml) was abrogated by neutralizing RANKL (with 2.5 µg/ml anti-RANKL antibody), and is similar to the response to the cytokine SDF-1α (100 ng/ml) (e). Invasion assays using 96-well chemotaxis chamber with polycarbonate filters (8 µm pore size) coated with human type I collagen showed that RANKL increases invasion of MDA-231BO2 human breast cancer cells in a dose-dependent manner (f, g). RANKL (1 µg/ml) had a similar effect to the cytokine SDF1α (100 ng/ml). Neutralized RANKL or siRNA mediated knockdown of RANK significantly decreased RANKL stimulation. siRNA mediated knockdown of MMP-1 impaired invasion in a similar level of cells treatment with the PI3K inhibitor wortmannin (100 nM) (h). All experiments were run in triplicate. Error bars represent variation between technical replicates, except for siRNA mediated knockdown of MMP-1 and RANK were it represents the average of three independent clones. n = 3, *p<0.05, **p<0.01, ***p<0.005, using one-way ANOVA with a Newman-Keuls multiple comparison test.

Next we analyzed the ability of RANKL, which does not affect cell proliferation (Fig. 2a), to stimulate the migration of RANK^+^ cell lines. Breast cancer cell lines show different basal migration ability, with MDA-231BO2 having the highest basal migration among breast cancer cell lines, similar to the prostate cancer cell line PC-3 (Fig. 2b). Breast cancer cell line MDA-231BO2 and the prostate cancer cell line PC-3, showed the highest response to RANKL in comparison to non stimulated cells (Fig. 2c). To determine that the increase in cell migration was RANK-dependent, a stable knockdown of RANK in the RANK^+^ prostate cancer cell line PC-3, that expresses the highest levels of RANK, was obtained (Fig. S1a). Migration induced by RANKL was significantly decreased in the PC-3^shRANK^ cells in comparison to RANK expressing cells (p<0.05) (Fig. 2c). The effect of RANKL on cell migration was dose-dependent (Fig. 2d), and significantly neutralized upon the addition of an anti-RANKL antibody that captures the soluble RANKL (p<0.05) (Fig. 2e). The stimulatory effect observed with RANKL was similar to the effect of stromal cell-derived factor 1-α (SDF-1α), a chemotatic factor that promotes breast cancer cells migration *in vitro* (Fig. 2e). RANK expression levels did not affected basal migration, since PC-3^shRANK^ and parental cells have similar basal migration levels (Fig. 2b). Regarding a potential autocrine activation of RANKL-RANK pathway, we did not observe expression of RANKL in the studied cell lines.

We then analyzed if RANKL could also affect the invasion of RANK^+^ tumor cells through a type I collagen matrix, using the breast cancer cell line MDA-231BO2. RANKL induced cell invasion in a dose-dependent manner (Fig. 2f, g). The increase in cell invasion in response to 1 µg/ml RANKL was about 70% when compared to untreated cells and, as it was previously observed for cell migration, the effect of RANKL was significantly neutralized by the addition of an anti-RANKL antibody (p<0.01), and was similar to the effect of SDF-1α (Fig. 2h). Using siRNA mediated transient knockdown of RANK in MDA-231BO2 cells (Fig. S1b), we observed a significant decrease in cell invasion upon RANKL stimulus (p<0.001) (Fig. 2h). We also analyzed the effect of the expression of the type I collagenase MMP-1 in cell invasion through the collagen matrix. MMP-1 transient knockdown (Fig. S1c) was sufficient to impair cell invasion and was not rescued by the stimulus with RANKL (p<0.001) (Fig. 2h). This effect was identical to the cell treatment with the migration (via PI3K) inhibitor wortmannin.

### Activation of RANKL-RANK pathway in breast cancer cells up-regulates MMP-1 expression

**Figure 3:**
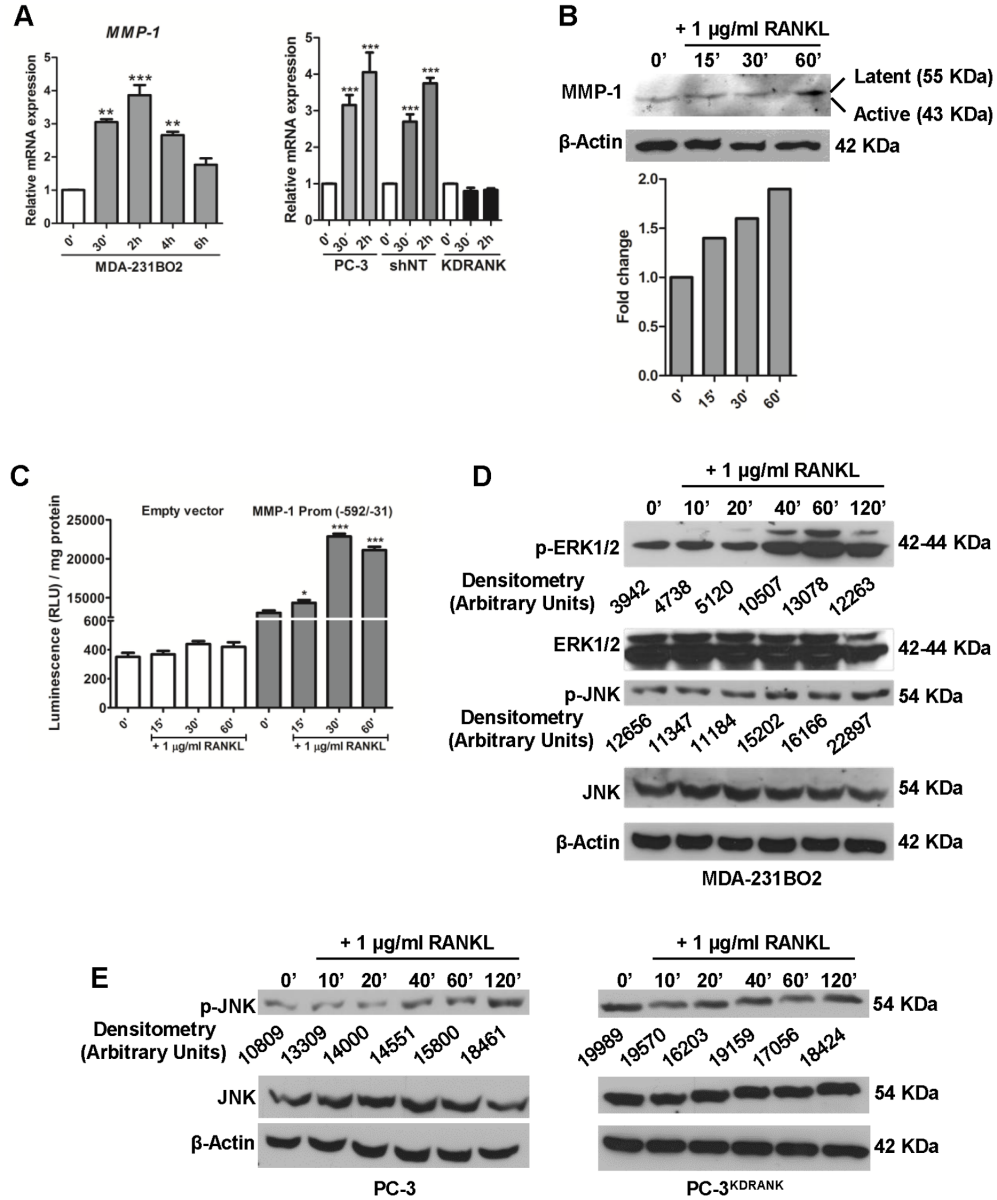
Activation of RANKL-RANK pathway up-regulates MMP-1 expression in breast cancer cells. *MMP-1* expression upon RANKL stimulus was analyzed by RT-qPCR. MDA-231BO2 breast cancer cells or PC-3 prostate cancer cells were cultured with 1 µg/ml RANKL and total RNA was extracted at different time points. *MMP-1* mRNA expression (mean ± SEM) was measured by RT-qPCR (n = 3) (a). MMP-1 expression at the protein level upon RANKL stimulus was analyzed at different time points by Western blot. MDA-231BO2 cells were cultured with 1 µg/ml RANKL for 60 min. Protein lysates from the treated cells were analyzed for MMP-1 protein expression. β-Actin was used as loading control (b). PC-3 prostate cancer cells were transfected with pGL4.15[l*uc2P*/hygro] plasmid containing the *MMP-1* gene promoter sequence (−592/−31). Cells were serum-starved for 24 h, and then treated with 1 µg/ml RANKL for 60 min before measuring luciferase activity. Results are expressed as the mean ± SEM (n = 3) of the relative luciferase activity (c). RANKL induces ERK1/ERK2 and JNK phosphorylation on MDA-231BO2 and PC-3 cells. RANK knockdown abrogated JNK phosphorylation. Cells were serum-starved for 24 h and stimulated with 1 µg/ml RANKL for the indicated time periods. ERK1/ERK2 activation (Thr202/Tyr204 phosphorylation; p-ERK), and JNK activation (Thr183/Tyr185; p-JNK) were detected by Western blot. Total ERK1/2, JNK, and control β-actin protein levels are shown. Phosphorylated protein levels, p-ERK1/2 and p-JNK, were normalized by densitometry to total protein levels, ERK1/2 and JNK (prior normalized to β-actin levels) (d,e). All experiments were run in triplicate. Error bars represent variation between technical replicates. n = 3, *p<0.05, **p<0.01, ***p<0.005, using one-way ANOVA with a Newman-Keuls multiple comparison test.

To evaluate if RANKL-RANK pathway activation in RANK^+^ breast and prostate cancer cells could alter MMP-1 levels, we analyzed MMP-1 expression at both mRNA and protein levels upon RANKL stimulus. MMP-1 is among the “bone metastasis signature” genes identified using human breast cancer cells in a mouse model [30], and found to be overexpressed in tumor cells of human bone metastases when compared to a human normal epithelial cell line [34]. We analyzed the expression of *MMP-1* (Fig. 3a), and *ADAMTS1*, *OPN*, *IL-11*, *CXCR4*, *CTGF*, and *PTHrP* (Fig. S2). *MMP-1*, *ADAMTS1*, *OPN*, and *PTHrP* were up-regulated in MDA-231BO2 breast cancer cells upon RANKL stimulus. RANK knockdown in PC-3 prostate cancer cells abrogated the effect of RANKL on *MMP-1* gene expression (Fig. 3a). At the protein level, MMP-1 also increased after RANKL stimulus (Fig. 3b).

To clarify if the *MMP-1* promoter was targeted by RANKL signaling, *MMP-1* promoter (−592/−31 region) was tested for RANKL responsiveness by luciferase assay (Fig. 3c). The results suggest that *MMP-1* is transcriptionally activated by RANKL. Since RANKL-RANK signaling pathway is thought to activate a downstream phosphorylation cascade, that can be involved in *MMP-1* gene transcription via AP-1, we next assessed the activation of ERK1/2 and JNK by RANKL in MDA-231BO2 cells. RANKL induced both ERK1/2 and JNK activation (Fig. 3d). RANK knockdown in PC-3 prostate cancer cells abrogated downstream activation of JNK (Fig. 3e).

### Knockdown of MMP-1 decreases osteolytic lesions and osteoclast recruitment to tumor-bone interface in a mouse model of bone metastases

**Figure 4:**
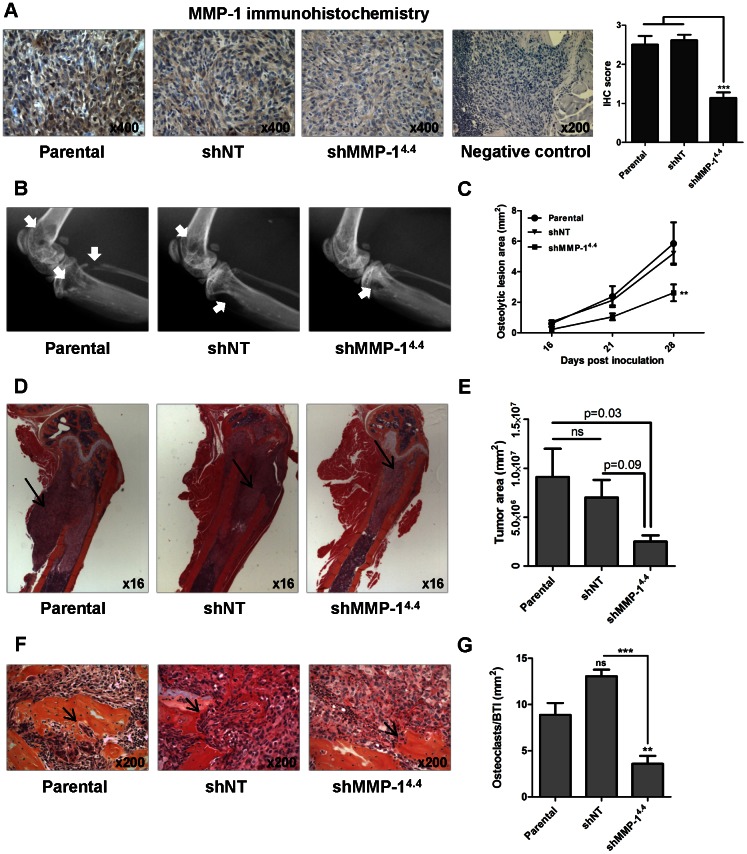
Knockdown of MMP-1 decreases osteolytic lesions and osteoclast recruitment to tumor-bone interface *in vivo*. Representative MMP-1 staining in demineralized bone sections from mice with bone metastases inoculated with MDA-231BO2 parental, shNT or shMMP1^4.4^ cells. MMP-1 expression was quantified according to stain intensity (0–3). Results are expressed as the mean ± SEM. ***p<0,005 with a one-way ANOVA with a Newman-Keuls multiple comparison test (n = 6–13 per group) (a). Representative x-ray images from hind limbs of mice 4 weeks post inoculation with MDA-231BO2 parental, shNT and shMMP-1 cells. Arrows indicate osteolytic lesions (b). Osteolytic lesion area measured on radiographs of hind limbs and forelimbs of mice with bone metastases. Results are expressed as the mean area ± SEM per mouse (n = 6–13 per group). ** p<0.01 compared to parental or shNT clones using a two-way ANOVA with a Bonferroni post-test at 4 weeks (c). Representative histology of femurs with tumor indicated by arrows (d). Tumor burden in hind limbs and forelimbs was measured by quantitative histomorphometry. Results are expressed as the mean ± SEM area per mouse. A one-way ANOVA with a Newman-Keuls multiple comparison test showed a significant difference between parental and shMMP1^4.4^ groups (p<0.05) but no significant differences between shNT and shMMP1^4.4^ groups (n = 6–13 per group) (e). Representative bone histology of the femurs. Osteoclasts are indicated by arrows (f). Osteoclast number was measured in the femur at 200× magnification. Results are expressed as the number of osteoclasts (OC) per mm^2^ bone-tumor interface (BTI). Results are expressed as the mean ± SEM OC/BTI (g). ns – no significant **p<0,01, ***p<0,005 with a one-way ANOVA with a Newman-Keuls multiple comparison test (n = 6–13 per group).

To assess if MMP-1 could have a major role in breast cancer-induced osteolytic lesions, we analyzed the effect of MMP-1 knockdown in bone osteolysis in a mouse model of breast cancer bone metastases. Mice were inoculated in the left ventricle with the parental cell line MDA- 231BO2, or with non-target (NT, scrumble) or MMP-1 stable knockdown clones (Fig. S1d, e). MMP-1 knockdown resulted in lower levels of MMP-1 expression in the metastatic tumors, as assessed by immunohistochemistry (Fig. 4a). The development of osteolytic lesions was significantly decreased in the mice inoculated the MDA- 231BO2^shMMP-1^ cells (p<0.01) as assessed by measuring osteolytic lesion area on x-ray (Fig. 4b, c).

We also performed a histomorphometric analysis on all the long bones to determine tumor burden upon mice sacrifice. We observed a tumor burden decrease in the mice inoculated the MDA-231BO2^shMMP-1^ cells (Fig. 4d, e). To clarify if osteoclastogenesis was involved in the observed decrease in osteolysis and eventually tumor burden, we quantified the osteoclast recruitment to the tumor-bone interface. It was significantly lower in the mice inoculated the MDA- 231BO2^shMMP-1^ cells (p<0.001) (Fig. 4f, g).

## Discussion

In this work we explore the role of MMP-1 in RANK+ breast and prostate cancer cells upon activation of RANKL-RANK pathway, and its ability to promote a metastatic behavior in these cells.

It was previously demonstrated that tumor cells can express RANK and activate RANKL-RANK pathway [13], [14], [15]. However, the relevance of RANK-positive tumor cells in the clinical setting is incompletely understood. Different studies report different percentages of positive samples, probably due to differences in the immunohistochemical detection of RANK [22], [23], [24], [25], [26], [27]. Also, so far there is insufficient evidence for a prognostic value of RANK in primary breast tumors [22], [27].

Here, we demonstrate that RANK is expressed by different breast and prostate cancer cell lines *in vitro*. Upon stimulation of MDA-231BO2 cells, a RANK+ human bone-seeking breast cancer cell line, RANKL activates specific downstream signaling pathways, namely JNK (c-Jun N-terminal kinases) and ERK1/2 (extracellular signal regulated kinase), leading to an invasive phenotype. In fact, these cells show increased migration and invasiveness. RANK knockdown in the PC-3 prostate cancer cell line, that expresses the highest levels of RANK, abrogated these effects.

RANKL stimulation induces the over expression of several genes implicated in bone metastasis [30], [34]. Among such genes are the bone metastasis-related collagenase *MMP-1* and *ADAMTS1*, which orchestrate a paracrine signaling cascade to modulate the bone microenvironment in favor of osteoclastogenesis and bone metastasis [31]. These two proteases cause the release of membrane bound epidermal growth factor (EGF)-like growth factors from tumor cells, suppressing the expression of OPG in osteoblasts and increasing osteoclast differentiation. Elevated MMP-1 and ADAMTS1 expression is associated with increased risk of bone metastasis in breast cancer patients. *In vivo*, MMP-1 was shown to be overexpressed in breast cancer samples, compared to normal breast tissue [35], and in bone metastasis from breast cancer, compared to brain metastases [36] or normal breast tissue [34]. Recently, MMP-1 and other bone metastasis-associated genes were evaluated as predictive biomarkers associated with breast cancer bone metastasis, and was highly expressed in both primary and metastatic breast cancer [37].

Since invasion through type I collagen matrix was increased upon RANKL stimulus we hypothesized that MMP-1 could play a major role in this cellular behavior. In fact, RANKL led to an increase in *MMP-1* expression at both mRNA and protein level, and our data showed that RANKL induced the MMP-1 promoter activity. As we hypothesized, knockdown of MMP-1 impaired invasion through type I collagen.

Next we aimed to define the relevance of MMP-1 expression in bone metastases *in vivo*. Using a mouse model of bone metastasis, we demonstrated that MMP-1 knockdown significantly decreased bone osteolysis and osteoclast recruitment to the tumor-bone interface, and also decreased tumor burden. These results are consistent with previous experiments in a murine model of bone invasion, where tumors expressing MMP-1 were associated with higher osteolysis [38]. We hypothesize that MMP-1 knockdown can decrease the availability of EGF-family ligands, increasing OPG expression, and thereby decreasing osteoclastogenesis [31].

In a previous study we found that the levels of type I collagen carboxy-terminal telopeptide (ICTP), generated by MMP-1 cleavage of type I collagen, increased significantly with disease progression in bone and were not influenced by bisphosphonate therapy in contrast to the N-telopeptide (NTx, an amino-acid epitope cleaved by osteoclastic cathepsin K activity), leading to the hypothesis that a mechanism that is not inhibited by bisphosphonates, therefore osteoclast-independent, can contribute to tumor-induced bone osteolysis [39]. In fact osteoclasts do not express MMP-1 [40]. In a study with breast cancer patients with bone metastasis we showed that ICTP and MMP-1 baseline levels were associated with a shorter time to development of skeletal-related events, suggesting that MMP-1 might be useful to detect patients with tumor-induced osteolysis [41]. More recently, in a study with 92 patients with bone metastasis from solid tumors, we demonstrated that MMP-1 was expressed in most cases, independently of tumor type, X-ray pattern of bone lesions, and previous bisphosphonate therapy. Serum MMP-1 levels were significantly associated with overall survival [32].

In conclusion, we demonstrate that invasive breast and prostate cancer cells can be functionally affected by the local microenvironment, and that specific factors like RANKL can exacerbate their metastatic potential. The possibility of targeting MMP-1 via RANKL inhibition may lead to better clinical outcomes. Moreover, additional studies clarifying the relevance of the RANKL-RANK-MMP-1 triad at the clinical level are mandatory.

## Materials and Methods

Detailed experimental procedures are described in Material and Methods S1.

### Ethics Statement

In all studies, mice were handled and euthanized in accordance with approved institutional, national and international guidelines. Animal protocols were approved by the Institution Animal Care and Use Committee at the University of Virginia and were in accordance with guidelines from the U.S. Public Health Service Policy on Humane Care and Use of Laboratory Animals and in compliance with the U.S. Animal Welfare Act.

### Cell culture, MTT assay, and gene knockdown

Normal human breast epithelial cells MCF10A (ATCC), MDA-MB-231 (ATCC), MCF-7 (ATCC), BO2/GFP.2 [33] and BO2f11 (MDA-231-BO2-Frt11) [42] breast cancer cell lines, and PC-3 prostate cancer cell line (ATCC) were cultured under standard conditions. Cell proliferation was determined by standard MTT assay. For transient knockdown of *MMP-1* or *RANK*, parental BO2/GPP.2 cells were transfected with siRNA specific vectors. For the preparation of stable clones, parental BO2f11 cells were transfected with shRNA vector against *MMP-1* and parental PC-3 prostate cancer cells were transfected with shRNA vector against *RANK* gene. Detailed description of knockdown experiments can be found in Material and Methods S1. Knockdown of *MMP-1* or *RANK* mRNA and protein were confirmed by RT-qPCR, and by Western blot analysis. Single stable clones were selected by limiting dilution in the presence of hygromycin B. MMP-1^KD^ and RANK^KD^ clones were retested for stability after culture in the absence of hygromycin B for 30 days. Two non-target controls (shNT) and two *MMP-1* knockdown (shMMP-1) clones were selected for *in vivo* experiments. *In vitro* cellular assays were performed with three different non-target or *MMP-1* and *RANK* knockdown clones.

### Migration and invasion assays

Migration of cancer cells was assessed using a 96-well chemotaxis chamber with polycarbonate filters (8 µm pore size). All cells were serum-starved for 24 h and resuspended in chemotaxis medium. Cells were stimulated with 1 µg/ml RANKL (neutralized or not with 2.5 µg/ml anti-hTRANCE/TNFSF11 antibody) or 100 ng/ml SDF-1α, for 6 h. Calcein stained cells were counted using a Zeiss Axiovert 200M fluorescence microscope, with 200× magnification, 4 fields per well. A second migration assay was performed with Oris Cell Migration Assay – Collagen I Coated plates according to manufacturer's instructions. Invasion of cells was assessed using a 96-well chemotaxis chamber with polycarbonate filters (8 µm pore size) coated with 1.5 mg/ml human type I collagen. After an incubation period of 24 h cells were fixed, stained with crystal violet and counted using a Leica DM2500 bright field microscope, with 200× magnification, 4 fields per well.

### RT-qPCR and Western blot

Cells were serum-starved for 24 h, then treated 1 µg/ml RANKL for 10, 20, 40, 60 or 120 min. Total RNA (500 ng per sample) was reverse transcribed, and the resulting cDNAs were amplified by PCR or semi-quantitative real-time PCR, using specific primers for *GADPH*, *MMP-1*, *ADAMTS1*, *PTHrP*, *OPN*, *IL11*, *CXCR4*, *CTGF*, *RANK* and *RANKL* (detailed information about the primers used can be found in Supporting Information). Target gene expression was normalized against the housekeeping *GADPH* gene, and data were analyzed using the ΔΔCt method. For Western blot analysis of protein expression or phosphorylation, cells were lysed in 2× SDS-loading buffer with protease and phosphatase inhibitors cocktails. Antibodies used include: p-ERK1/2(Thr202/Tyr204), ERK1/2, p-JNK (Thr183/Tyr185), JNK, p-NFκB(Ser-536) from Santa Cruz; MMP-1 IM-35 from Calbiochem; and hRANK N-2B10 from Amgen. β-actin was used as control.

### Luciferase reporter assay

PC-3 prostate cancer cells were transfected with pGL4.15[l*uc2P*/hygro] constructs containing different *MMP-1* gene promoter sequences. Detailed description of transfection experiments can be found in Material and Methods S1. All inserts were confirmed by sequencing. Stable transfection was obtained by selection with 50 µg/ml hygromicin B. Cells were serum-starved for 24 h, then treated 1 µg/ml RANKL for 15 or 60 min. Cells were analyzed for luciferase activity using the Luciferase Assay System.

### Bone metastasis model and immunohistochemistry

Detailed description of animal experiments can be found in Material and Methods S1. Briefly, intracardiac inoculation of tumor cells was performed as previously described [43]. Osteolytic lesions were analyzed by radiography at 1, 2 3 and 4 weeks post tumor inoculation. Osteolytic lesion area was quantified using MetaMorph software. Forelimbs, hind limbs, and spine of the mice were collected 28 days after tumor inoculation, fixed, decalcified and processed. Longitudinal, midsagittal sections 3.5 µm in thickness from the tibia, femur and lumbar spines were stained with hematoxylin and eosin (H&E) and prepared for histomorphometric analysis. All sections were viewed on a Leica DM LB compound microscope with a Q-Imaging Micropublisher Cooled CCD color digital camera. Images were captured and analyzed using MetaMorph software. Tumor burden per bone, defined as area of bone occupied by the cancer cells, was calculated at the tibia, femur and humerus at 50× magnification on H&E stained section. Bone area was calculated at the tibia, femur and humerus at 16× magnification on H&E stained sections, for 5 mm of tissue, starting at the articular surface. Osteoclast number at the tumor-bone interface (OC/mm bone surface) in the femur, tibia and humerus was measured on H&E stained slides at 200× magnification.

Immunohistochemical analysis was performed on decalcified formol-fixed paraffin-embedded (FFPE) tissue sections using the mouse monoclonal anti-MMP-1 antibody. Immunostaining was scored on triplicate tissues using the following arbitrary scale: 0, no staining; 1, weak staining; 2, medium staining; 3, strong staining.

### Statistical analysis

*In vitro* data were analyzed with the use of Graphpad Prism v5.0 software. Samples were analyzed in triplicate for proliferation, migration, invasion, RT-qPCR and luciferase assays. Statistics were analyzed by unpaired t-test or one-way ANOVA and Newman-Keuls multiple comparison test. Results are expressed as mean ± SEM and p<0.05 was considered significant.

*In vivo* data were analyzed with the use of Graphpad Prism v5.0 software. Differences in osteolytic lesion area between clones and treatment groups were analyzed by two-way ANOVA. Histomorphometry for tumor burden and osteoclast number was analyzed by one-way ANOVA and Newman-Keuls multiple comparison test. All the results were expressed as mean±SEM, and p<0.05 was considered significant.

## Supporting Information

Figure S1 **RANK and MMP-1 stable knockdown.** Gene shRNA mediated knockdown of RANK in PC-3 prostate cancer cells was confirmed by RT-qPCR and Western blot (a). Gene siRNA mediated knockdown of RANK (b) and MMP-1 (c) in MDA-231BO2 breast cancer cells was confirmed by RT-PCR. Gene shRNA mediated knockdown of MMP-1 in MDA-231BO2 breast cancer cells was analyzed by RT-PCR (d) and retested by Western blot for stability after culture in the absence of hygromycin B for 30 days (e).
(TIFF)

Figure S2 **Activation of RANKL-RANK pathway up-regulates several bone metastasis-signature genes in breast cancer cells.** Gene expression upon RANKL stimulus was analyzed by RT-qPCR. MDA-231BO2 breast cancer cells were cultured with 1 µg/ml RANKL and total RNA was extracted at different time points. n = 3 (mean ± SEM).
(TIFF)

Material and Methods S1 **Detailed material and methods section.**
(DOCX)
