# Supplementary figures and images for: RANKL/RANK/MMP-1 Molecular Triad Contributes to the Metastatic Phenotype of Breast and Prostate Cancer Cells In Vitro
Source: PLoS One. 2013 May 16;8(5):e63153. doi: 10.1371/journal.pone.0063153 (PMC3656033; doi:10.1371/journal.pone.0063153)

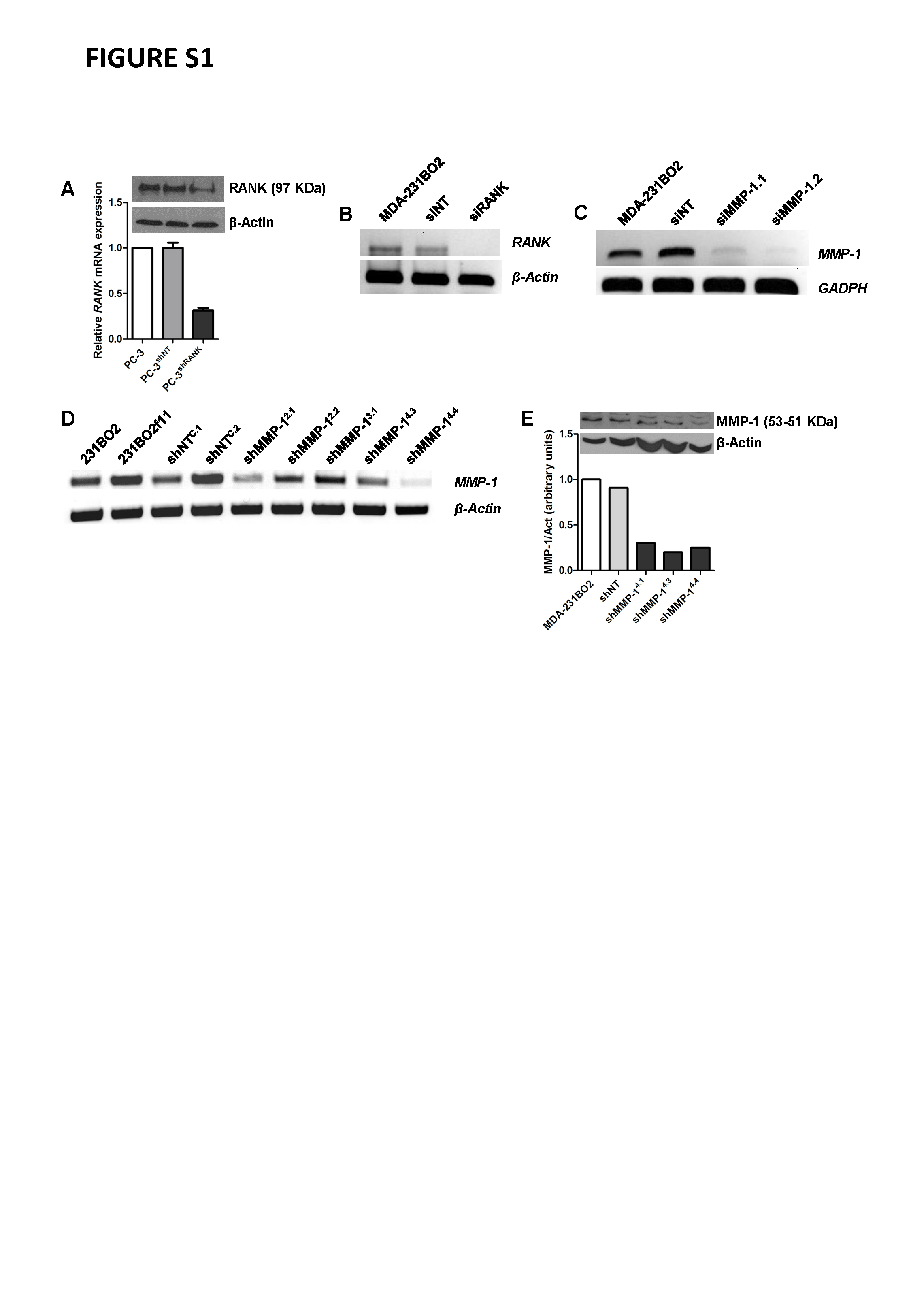

Supplement: Figure S1 — RANK and MMP-1 stable knockdown. Gene shRNA mediated knockdown of RANK in PC-3 prostate cancer cells was confirmed by RT-qPCR and Western blot (a). Gene siRNA mediated knockdown of RANK (b) and MMP-1 (c) in MDA-231BO2 breast cancer cells was confirmed by RT-PCR. Gene shRNA mediated knockdown of MMP-1 in MDA-231BO2 breast cancer cells was analyzed by RT-PCR (d) and retested by Western blot for stability after culture in the absence of hygromycin B for 30 days (e). (TIFF) [file pone.0063153.s001.tiff]

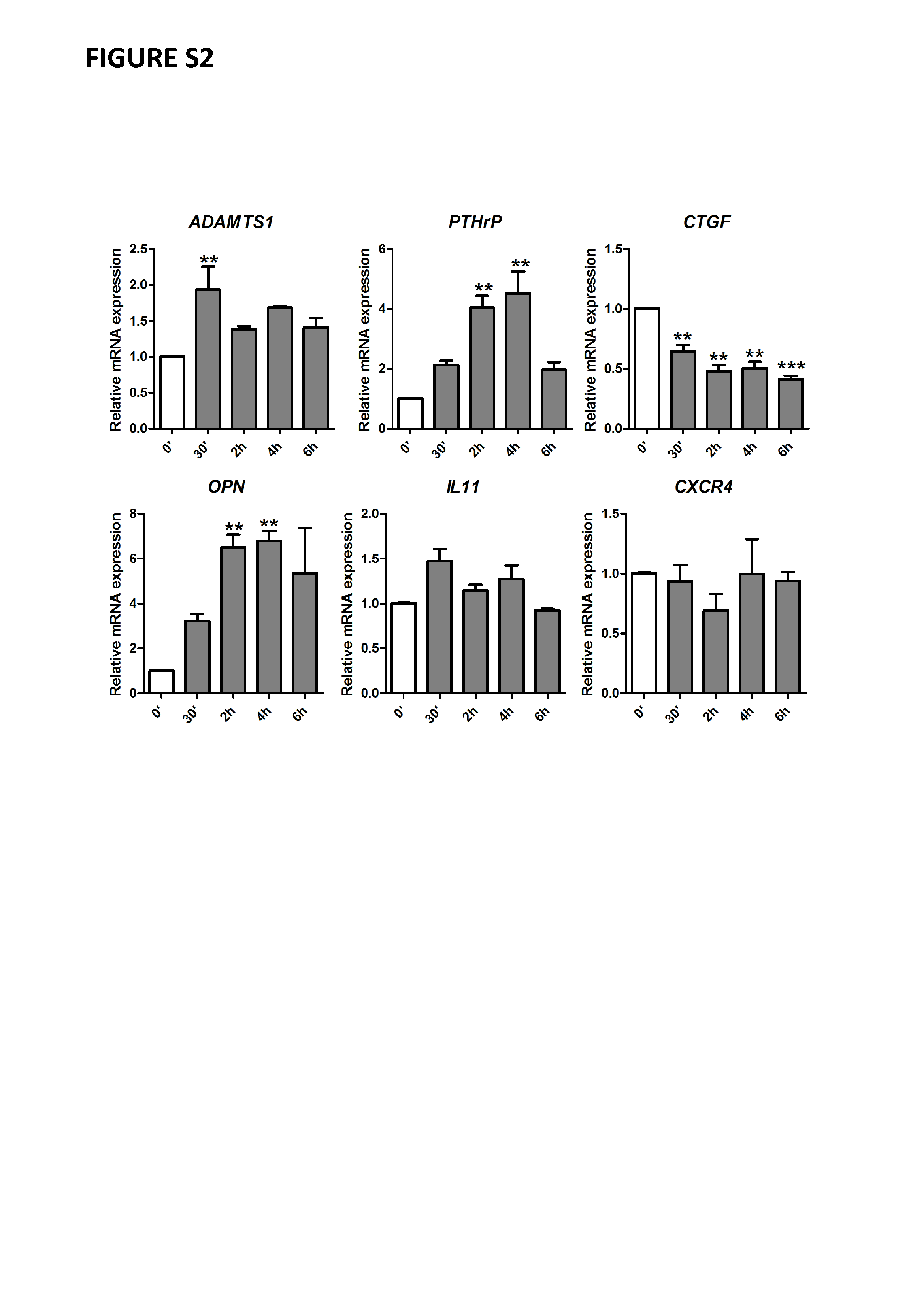

Supplement: Figure S2 — Activation of RANKL-RANK pathway up-regulates several bone metastasis-signature genes in breast cancer cells. Gene expression upon RANKL stimulus was analyzed by RT-qPCR. MDA-231BO2 breast cancer cells were cultured with 1 µg/ml RANKL and total RNA was extracted at different time points. n = 3 (mean ± SEM). (TIFF) [file pone.0063153.s002.tiff]
