# Supplementary material for: RANKL/RANK/MMP-1 Molecular Triad Contributes to the Metastatic Phenotype of Breast and Prostate Cancer Cells In Vitro
Source: PLoS One. 2013 May 16;8(5):e63153. doi: 10.1371/journal.pone.0063153 (PMC3656033; doi:10.1371/journal.pone.0063153)
Supplement: Material and Methods S1 — Detailed material and methods section. (DOCX) [file pone.0063153.s003.docx]

**Material and Methods S1**

**Cell culture, MTT assay, and gene knockdown**

MCF10A (CRL-10317, ATCC) normal mammary epithelial cells were cultured in mammary epithelial cell growth medium (MEGM) medium (Clonetics) supplemented with 0.4% bovine pituitary extract, 0.1% hEGF, 0.15 hydrocortisone, 0.1% insulin, and 100 ng/ml cholera toxin (Sigma); MDA-MB-231 (HTB-26, ATCC) breast cancer cells were cultured in Dulbecco’s modified Eagle’s medium (DMEM) (Gibco, Invitrogen) containing 10% fetal bovine serum (FBS) (Gibco) and 1% penicillin/streptomycin (Gibco). BO2/GFP.2 [40] and BO2f11 breast cancer cells (MDA-231-BO2-Frt11) [42] were cultured in RPMI1640 medium (Gibco) supplemented with 10% fetal bovine serum, 0.25 mg/ml G418 (Invitrogen) and 1% penicillin/streptomycin. PC-3 prostate cancer cells (CRL-1435, ATCC) were cultured in RPMI1640 medium supplemented with 10% FBS and 1% penicillin/streptomycin. MCF-7 breast cancer cells (HTB-22, ATCC) were cultured in EMEM medium (Gibco) supplemented with 10% FBS, 2 mM L-Glutamine (Gibco), 1 mM sodium pyruvate (Gibco), 0.1 mM non-essential aminoacids (Gibco), 0.01 mg/ml bovine insulin (Gibco) and 1% penicillin/streptomycin. Cells were maintained at 37ºC with 5% CO2 in a humidified chamber.

Cell proliferation was assayed by MTT assay. Cells were plated in 96-well plates (2x10^3^ cells/well). At approximately 80% confluence, cells were serum starved for 24h in culture medium supplemented with 0.1% FBS. Cells were stimulated for 24h with 500 ng/ml to 2.5 µg/ml RANKL (Amgen). Medium was replaced by 200 µl fresh medium and MTT reagent (50 µl, 5 mg/ml) (Sigma-Aldrich) was added to each well. After a 4 h-incubation at 37ºC, medium was replaced by 200 µl DMSO (Sigma-Aldrich) and 25 µl glycine buffer. Absorbance was measured at 550 nm using an InfiniteM200 spectrophotometer (Tecan).

For the preparation of transient clones, parental MDA-231BO2 cells were transfected with siRNA against *MMP-1* or *RANK* genes (Santa Cruz) using siRNA Transfection Reagent (Santa Cruz), according to manufacturer’s instructions. Cells transfected with a non-target siRNA were used as a control. Knockdown of *MMP-1* or *RANK* mRNA and protein were confirmed by RT-qPCR and Western blot analysis. For the preparation of stable clones, parental MDA-MB-231BO2f11 cells were transfected with shRNA vector against *MMP-1* and parental PC-3 prostate cancer cells were transfected with shRNA vector against *RANK* gene (SA Biosciences) using SureFECT Transfection Reagent (SA Biosciences), according to manufacturer’s instructions. Cells transfected with a non-target shRNA were used as a control in both cases. Knockdown of *MMP-1* or *RANK* mRNA and protein were confirmed by RT-qPCR and Western blot analysis. Single clones were selected by limiting dilution in the presence of hygromycin B (Sigma). MMP-1^KD^ and RANK^KD^ clones were retested for stability after culture in the absence of hygromycin B for 30 days. Two nontarget controls (shNT) and two *RANK* or *MMP-1* knockdown (shMMP-1) clones were selected for *in vivo* and *in vitro* experiments.

**Migration and invasion assays**

Migration of cancer cells was assessed using a 96-well chemotaxis chamber (Receptor Technologies) with polycarbonate filters (8 µm pore size). All cells were starved for 24 h in culture medium with 0.1% FBS, and resuspended in chemotaxis medium (DMEM supplemented with 0.1% FBS and 12 mM HEPES (Gibco, Invitrogen) to 4x10^5^ cells/ml. 1 µg/ml RANKL (neutralized or not by 1h incubation at 37ºC with 2 µg/ml anti-hTRANCE/TNFSF11 antibody, R&D Systems) or 100 ng/ml SDF-1α (R&D Systems) were placed in the lower wells and 2x10^4^ human cancer cells were placed in the upper wells. After an incubation period of 6h at 37ºC with 5% CO2, the medium in the upper well was replaced by 2 mM EDTA, and plates were incubated 30 min at 4ºC and spinned at 400 g for 10 min. Medium in lower wells was replaced by 5 µg/ml Calcein AM (Calbiochem, Merck) and plates were incubated for 1h at 37ºC with 5% CO2. Cells were counted using a Zeiss Axiovert 200M fluorescence microscope (Zeiss), with 200x magnification, 4 fields per well.

A second migration assay was performed with Oris Cell Migration Assay – Collagen I Coated plates (Platypus Technologies) according to manufacturer’s instructions. Cells were plated at a density of 2.5x10^5^ cells/ml, were allowed to adhere for 6h, and to migrate for 18h. After Calcein AM staining relative fluorescence was read in an InfiniteM200 spectrophotometer (Tecan).

Invasion of cells was assessed using a 96-well chemotaxis chamber (Receptor Technologies) with polycarbonate filters (8 µm pore size) coated with 1.5 mg/ml human type I collagen (StemCell Technologies). All cells were starved for 24 h in culture medium with 0.1% FBS, and resuspended in invasion medium (DMEM supplemented with 0.1% FBS and 12 mM HEPES) to 4x10^5^ cells/ml. RANKL (neutralized or not by 1h incubation at 37ºC with 2 µg/ml anti-hTRANCE/TNFSF11 antibody) or the chemokine SDF-1α were placed in the lower wells and 2x10^4^ human cancer cells were placed in the upper wells. For PI3K inhibition cells were pretreated by 1h incubation with 100 nM wortmannin (Sigma-Aldrich). After an incubation period of 24h at 37ºC with 5% CO2, the medium in the upper well was removed and cells in the lower surface of polycarbonate filter were fixed with 2.34% PFA for 10 min and stained with crystal violet (Sigma-Aldrich) for 10 min at room temperature. Cells were swabbed from the upper surface of the filter and invading cells were counted using a Leica DM2500 bright field microscope (Leica), with 200x magnification, 4 fields per well.

**RT-qPCR and Western blot**

Cells were rinsed in 1x PBS and RNeasy mini spin column (Qiagen) were used for total RNA isolation according to manufacturer’s instructions. DNase I (Promega) treatment was performed to remove genomic DNA contamination and RNA concentration and purity was assessed in a NanoDrop spectrophotometer (Thermo Fisher Scientific). RNA (500 ng per sample) was reverse transcribed using Superscript III First-Strand Synthesis System for RT-PCR (Invitrogen) according to the manufacturer’s instructions with anchored oligo(dT) primer. cDNAs were amplified by semi-quantitative real-time PCR (qPCR) using RT^2^ SYBR Green qPCR Master Mix (SABiosciences, Quiagen) and specific primers for *GADPH* (PPH00150E), *MMP-1* (PPH00120B), *ADAMTS1* (PPH01149A)*, PTHrP* (PPH02141A)*, OPN* (PPH00582E)*, IL11* (PPH00573D)*, CXCR4* (PPH00621A)*, CTGF* (PPH00550F), *RANK* (PPH01102B) and *RANKL* (PPH01048E) (RT^2^ qPCR Primer Assay, SABiosciences, Quiagen), in a Rotor Gene 6000 (Corbett, Quiagen), for 40 cycles (95ºC for 15 s, 55ºC for 40 s, 72ºC for 30 s) after an initial incubation at 95ºC for 10 min. Reactions were performed in triplicate. Target gene expression was normalized against the housekeeping gene *GADPH*, using the mean value of the three replicates.

For Western blot analysis of protein expression or phosphorylation, cells were seeded in 35 mm diameter plates (10^5^ cells/well). At approximately 80% confluence cells were serum starved overnight in culture medium supplemented with 0.1% FBS, then treated with 1 µg/ml RANKL for 10, 20, 40, 60 or 120 min. Cells were washed once with PBS, lysed in 200 µl 2x SDS-loading buffer with protease and phosphatase inhibitors cocktails (Sigma-Aldrich), and heated to 95ºC for 10 min. Samples were loaded onto a 10% polyacrylamide gel and electrophoresis was performed using a Mini-PROTEAN Tetra cell (BioRad). Proteins were transferred onto a Protran BA85 nitrocellulose membrane (Whatman) using a Mini-PROTEAN Tetra Cell transfer system (BioRad). Membranes were blocked in PBST, 5% skim milk for 1 h, incubated overnight with the primary antibody and for 2h with the secondary antibody. Antibody detection was performed using SuperSignal West Pico Chemiluminescent HRP Substrate (Pierce) according to the manufacturer’s directions and signal was visualized on radiographic film. Antibodies used include: p-ERK1/2(Thr202/Tyr204), ERK1/2, p-JNK (Thr183/Tyr185), JNK, p-NFκB(Ser-536) from Santa Cruz; MMP-1 IM-35 from Calbiochem; hRANK N-2B10 from Amgen; β-actin (Abcam) was used as control. Anti-mouse IgG and anti-rabbit IgG secondary antibodies conjugated to peroxidase were purchased from Santa Cruz.

**Luciferase reporter assay**

PC-3 prostate cancer cells were transfected with pGL4.15[l*uc2P*/hygro] constructs containing different *MMP-1* gene promoter sequences using SureFECT Transfection Reagent (SA Biosciences, Quiagen), according to manufacturer’s instructions. Stable transfection was obtained by selection with 50 µg/ml hygromicin B (Sigma-Aldrich). Cells were seeded in 35 mm diameter plates (10^5^ cells/well). At approximately 80% confluence, cells were serum-starved for 24h in medium supplemented with 0.1% FBS, then treated 1 µg/ml RANKL for 15 or 60 min. Cells were analyzed for luciferase activity using the Luciferase Assay System (Promega), according to the manufacturer’s instructions on an InfiniteM200 luminometer (Tecan). MMP-1 promoter (region -592 to -31) was amplified by PCR using GoTaq Flexi DNA polymerase (Promega). Products was digested overnight with HindIII and XhoI (Promega), purified on agarose gel with the QIAquick Gel Extraction kit (Quiagen) according to the manufacturer’s instructions, and ligated into the pGL4.15[l*uc2P*/hygro] vector using T4 DNA ligase (Promega) according to the manufacturer’s instructions. All inserts were confirmed by sequencing.

**Bone metastasis animal model and immunohistochemistry**

Animal protocols were approved by the Institution Animal Care and Use Committee at the University of Virginia and were in accordance with guidelines from the U.S. Public Health Service Policy on Humane Care and Use of Laboratory Animals and in compliance with the U.S. Animal Welfare Act.

Female athymic nude mice, 4 weeks of age, were housed under barrier conditions in laminar flow isolated hoods. Autoclaved water and mouse chow were provided *ad libitum*. Animals bearing human tumor xenografts were carefully monitored for established signs of distress and discomfort and were humanely euthanized when these were confirmed. Intracardiac inoculation of tumor cells was performed as previously described [41]. Briefly, tumor cells were trypsinized, washed twice and resuspended in PBS to a final concentration of 10^6^ cells/ml. Animals were anesthetized with ketamine/xylazine and positioned ventral side up. MDA-MB-231 parental or clonally-derived cells were inoculated into the left ventricle by percutaneous injection using a 26-gauge needle. Osteolytic lesions were analyzed by radiography at 1, 2 3 and 4 weeks post tumor inoculation, using a Faxitron MX-20 with digital camera (Faxitron X-ray Corporation). Mice were imaged in a prone position at 1x magnification and 4x when osteolytic lesions were suspected. Osteolytic lesion area was quantified using MetaMorph software (Universal Imaging Corporation).

Forelimbs, hind limbs, and spine of the mice were collected 28 days after tumor inoculation, upon euthanasia, and fixed in 10% neutral buffered formalin for 48 h and decalcified in 10% EDTA for 2 weeks. After decalcification tissues were processed in Shandon Excelsior automated tissue processor (Thermo Fisher Scientific) and embedded in paraffin wax for sectioning. Longitudinal, midsagittal sections 3.5 µm in thickness from the tibia, femur and lumbar spines were cut using an automated Microm HM 355 S microtome (Thermo Fisher Scientific). Tissue sections were stained with hematoxylin and eosin (H&E) and prepared for histomorphometric analysis. All sections were viewed on a Leica DM LB compound microscope (Leica Microsystems) with a Q-Imaging Micropublisher Cooled CCD color digital camera (Vashaw Scientific Inc.). Images were captured and analyzed using MetaMorph software. Tumor burden per bone, defined as area of bone occupied by the cancer cells, was calculated at the tibia, femur and humerus at 50x magnification on H&E stained section. Bone area was calculated at the tibia, femur and humerus at 16x magnification on H&E stained sections, for 5mm of tissue, starting at the articular surface. Osteoclast number at the tumor-bone interface (OC/mm bone surface) in the femur, tibia and humerus was measured on H&E stained slides at 200x magnification.

Immunohistochemical analysis was performed on decalcified formol-fixed paraffin-embedded (FFPE) tissue sections. Paraffin sections (5-µm thick) were treated in xylene and rehydrated in a gradient of ethanol. For MMP-1 detection, endogenous peroxidase was blocked by 15 min incubation with 3% H_2_O_2_ in methanol, in the dark. Endogenous protein was blocked with Protein Block Serum-free (Dako) for 1h. Sections were incubated with a mouse monoclonal anti-MMP-1 antibody (IM-35, Calbiochem) for 1 h, followed by 30 min incubation with biotinylated anti-mouse IgG antibody. After a 30 min incubation with ABC Elite kit (Vector Laboratories) according to manufacturer’s instructions, slides were incubated with 3,3’-diaminobenzidine (DAKO), and counterstained with haematoxylin. Immunostaining was scored on triplicate tissues using the following arbitrary scale: 0, no staining; 1, weak staining; 2, medium staining; 3, strong staining.

**Statistical analysis**

*In vitro* data were analyzed with the use of Graphpad Prism v5.0 software. Samples were analyzed in triplicate for proliferation, migration, invasion, RT-qPCR and luciferase assays. Statistics were analyzed by unpaired t-test or one-way ANOVA and Newman-Keuls multiple comparison test. Results are expressed as mean±SEM and p<0.05 was considered significant.

*In vivo* data were analyzed with the use of Graphpad Prism v5.0 software (GraphPad Software). Differences in osteolytic lesion area between clones and treatment groups were analyzed by two-way ANOVA. Histomorphometry for tumor burden and osteoclast number was analyzed by one-way ANOVA and Newman-Keuls multiple comparison test. All the results were expressed as mean±SEM, and p<0.05 was considered significant.
